# Grass Rhizome Proteomics Reveals Convergent Freezing-Tolerance Strategies

**DOI:** 10.1101/2025.05.15.654294

**Published:** 2025-05-19

**Authors:** Elad Oren, Jingjing Zhai, Travis E Rooney, Ruthie Angelovici, Charles O. Hale, Lara J. Brindisi, Sheng-Kai Hsu, Christine M. Gault, Jian Hua, Thuy La, Nicholas Lepak, Qin Fu, Edward S. Buckler, M. Cinta Romay

**Affiliations:** 1 Institute for Genomic Diversity, Cornell University, Ithaca, NY 14853, USA; 2 Sesaco Corporation, Austin, TX 78701, USA; 3 Division of Biological Sciences and Interdisciplinary Plant Group, University of Missouri, Columbia, Missouri, 65211, USA; 4 School of Integrative Plant Sciences, Plant Breeding and Genetics Section, Cornell University, Ithaca, New York, 14853, USA; 5 School of Integrative Plant Science, Plant Biology Section, Cornell University, Ithaca, New York, 14853, USA; 6 USDA-ARS, Ithaca, NY, USA 14853; 7 Proteomics and Metabolomics Facility, Cornell University, Ithaca, New York, 14850, USA

**Keywords:** Grass, Tripsacum, Freezing tolerance, Proteomics

## Abstract

Early maize planting requires cold tolerance in temperate regions, which elite maize lacks. Extending the growing season could boost productivity along with more efficient nutrient use. Wild PACMAD grasses have each independently evolved freezing tolerance. To uncover the molecular basis of this convergence, we performed tandem mass tag labeling with shotgun mass spectrometry to measure protein abundance in rhizome tissues sampled during winter dormancy and summer activity. Our study examined five species: *Panicum virgatum*, *Andropogon gerardii*, *Miscanthus giganteus*, *Sorghastrum nutans* and hybrids of *Tripsacum floridanum* and *Tripsacum dactyloides* (maize’s sister genus), all grown in Ithaca, NY (where winter lows reach −29 °C). Of ~3500 proteins per species, 330 families showed consistent upregulation in winter, but only three—late embryogenesis abundant 3 (LEA3), aldose reductase, and phosphatidylethanolamine‐binding protein (PEBP)—were shared across all five lineages. LEA3 proteins display conserved hydrophobicity patterns in cold-tolerant species that are disrupted in maize. Functional enrichment highlighted recurrent use of lipid transfer proteins, heat shock proteins and other drought‐associated cryoprotectants. We next explored whether these conserved signatures extended to other tissues within *Tripsacum* and their orthologous genes in cold-sensitive maize. Comparisons with mRNA studies revealed that rhizome proteomic responses more closely resemble those of seedling leaves than roots, consistent with their shoot-derived anatomical identity, a pattern less pronounced in maize seedlings. Overall, the independent evolution of rhizome frost tolerance in PACMAD grasses appears to be governed by similar mechanisms driven primarily by expression‐level changes complemented by protein structural adaptations offering candidate targets for improving freezing tolerance in maize.

## Introduction

Maize (*Zea mays* ssp. mays), a vital crop for global food security, is susceptible to cold stress due to its tropical origins, limiting its yield in temperate regions. Early planting of maize can improve nitrogen capture, net primary productivity, and yield (Ojeda-Rivera et al. in press). However, this strategy requires cold tolerance, which is lacking in current elite maize varieties (Lyons 1973; Aroca et al. 2012). Cold tolerance has evolved multiple times within the *Panicoideae*, *Aristidoideae*, *Chloridoideae*, *Micrairoideae*, *Aristavideae, and Danthonioideae* (PACMAD) clade, a major lineage of predominantly C4 grasses (*Poaceae*) that diverged ~64 Mya, with ancestral roots in the Afrotropics (Sage et al. 2011; Gallaher et al. 2022). Thus, providing a valuable evolutionary resource for identifying core mechanisms underlying adaptation to cold and freezing adaptation. Among these, *Tripsacum*, the closest perennial and cold-tolerant relative of maize, having diverged about 650 Kya (Chen et al. 2022), is valuable for enhancing maize cold tolerance.

PACMAD grasses originated in warm-season climates unlike temperate diversified *Pooideae* and *Danthonioideae* (Kellogg 2015). However, most transitions of these grasses to colder climates occurred in tropical mountains, where cold-adapted lineages converged with temperate migrants (Schubert et al. 2020). Each PACMAD genus exhibits distinct evolutionary trajectories, polyploid histories, and biogeographic origins, having independently evolved robust cold and freezing tolerance over the past 20–30 million years (Gallaher et al. 2022; Stitzer et al. 2025).

Plant exposure to cold stress, including chilling (>0°C) and freezing (<0°C) temperatures, triggers physiological and molecular responses. Chilling stress disrupts membrane fluidity, impairs enzyme activity, and inhibits photosynthesis (Aroca et al. 2012), whereas freezing stress induces ice crystal formation in extracellular spaces, leading to cellular dehydration, membrane damage, and ultimately, cell death (Pearce 2001). Many temperate plants undergo cold acclimation. Adaptations resulting from cold acclimation include membrane lipid modifications (Degenkolbe et al. 2012; Barnes et al. 2016), accumulation of compatible solutes (Koster and Lynch 1992), enhanced antioxidant enzyme activity (Suzuki and Mittler 2006) and protective protein accumulation (Puhakainen et al. 2004; Xu et al. 2022). Plant cold perception involves plasma membrane modifications, including fluidity changes, Ca^2+^ channel activation, and cytosolic acidification, driving lipid and transcriptomic responses (Mori et al. 2018; Barnes et al. 2023). A widely conserved mechanism underpinning freezing tolerance is the activation of the C-repeat binding factor/dehydration-responsive element-binding (CBF/DREB) signaling pathway, which induces expression of cold-responsive (COR) genes (Thomashow 1999; Nakashima and Yamaguchi-Shinozaki 2006).

Studies in PACMAD grasses reveal both broadly conserved and species-specific molecular adaptations associated with this pathway. *Panicum virgatum* utilizes a zinc finger transcription factor, PvC3H72, to enhance ICE1-CBF-COR signaling, significantly increasing cold resilience (Xie et al. 2019). Additionally, calcium signaling pathways involving calmodulin and MEKK1 provide early response to cold stress (Ranaweera et al. 2022; Wu et al. 2024). *Pennisetum giganteum* maintains membrane fluidity at low temperature through the accumulation of unsaturated fatty acids such as linoleic and α-linolenic acids (Zhang et al. 2017; Li et al. 2020). Protective proteins including late embryogenesis abundant (LEA) proteins, dehydrins, and antioxidant enzymes are upregulated in response to freezing conditions, reducing oxidative injury and cellular dehydration (Liu et al. 2016; Xie et al. 2019). Species-specific adaptations include enhanced proline biosynthesis via the ornithine pathway in *Sorghum bicolor* (Vera-Hernández et al. 2018) and elevated activity of phenylpropanoid pathway enzymes (PAL and CAD) in *Miscanthus* to protect cells during freezing stress (Domon et al. 2013).

While previous studies in PACMAD grasses have elucidated cold adaptations primarily through transcriptomic and metabolomic analyzes, proteomic responses remain largely unexplored. Proteins, as the functional products of genes, provide direct evidence of cellular activity integrating transcriptional, translational, and post-translational regulation. Given the persistent nature of cold stress, proteomic analysis uniquely captures the accumulation of proteins reflecting acclimation and sustained adaptive responses. Furthermore, (Cope et al. 2025) demonstrated that proteins face stronger evolutionary constraints than transcripts, with protein abundances being highly conserved across species while mRNA levels can vary without substantially affecting protein output. This suggests that examining proteins directly is essential for understanding adaptive processes such as those underlying cold or freezing tolerance. Our multi-species study examines rhizome protein accumulation across seasonal variation to identify conserved freezing-tolerance mechanisms in PACMAD grasses, testing the hypothesis of genetic convergence where similar selective pressures drive parallel proteomic responses in distinct temperate grasses despite their divergent evolutionary histories.

## Results

**Figure 1.**
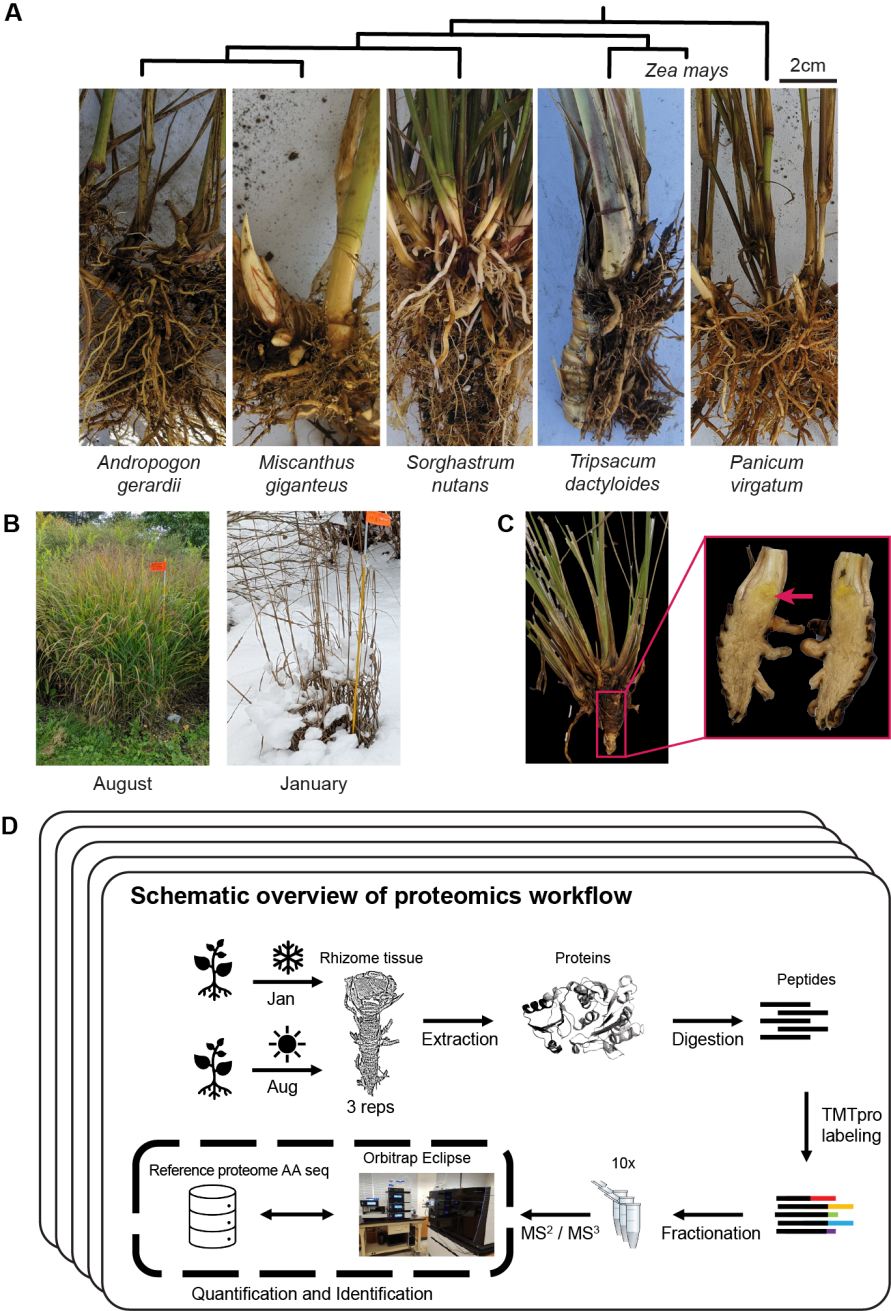
Overview of sampling and proteomics workflow. **(A)** Rhizomes from the five species sampled in the study: *Tripsacum spp*, *Panicum virgatum*, *Andropogon gerardii*, *Miscanthus giganteus*, and *Sorghastrum nutans*. Phylogeny of species presented along with *Z. mays* for reference **(B)** Field conditions during the two sampling seasons, illustrating the active growth in August and dormancy in January. **(C)** Example of the rhizome tissue sampled for proteomics, shown here for *Tripsacum*. The red arrow highlights the specific region collected. **(D)** Schematic overview of the proteomics workflow. Rhizome samples were collected each season, followed by protein extraction, digestion, and TMTpro labeling. Peptides were fractionated and analyzed via RTS-SPS-MS^3^ using an Orbitrap Eclipse mass spectrometer. Each species’ reference proteome was used for peptide and protein assignment, enabling quantification and identification.

To examine seasonal responses in PACMAD grasses, we analyzed rhizome tissues of five species using a proteomic shotgun approach during the winter (January) and summer (August) of 2019 and 2022 (Figure 1). The study site in Ithaca, New York (USDA Hardiness Zone 6a) experiences natural seasonal fluctuations, with an average winter temperature of −5°C (lows reaching −29°C) and summer temperatures averaging 20°C (highs of 26°C) over the five years preceding sampling (Northeast Regional Climate Center, 2025). The exposure to deep-freezing winters ensured that the proteomic responses captured were reflective of natural seasonal variation (Figure 1B).

Each season we sampled 2–3 replicated clones of *Sorghastrum nutans*, *Miscanthus giganteus*, *Andropogon gerardii*, and *Panicum virgatum* from established accessions at Cornell’s Botanical Gardens. For *Tripsacum*, we conducted more extensive sampling in 2019 (8 samples per season) and 2022 (24 samples per season) from seven-year-old hybrid clones from crosses involving *T. dactyloides* and *T. floridanum* at Cornell University’s Caldwell Field (Table 1).

Protein abundance analysis was performed in batches (see Methods). Across all five species a total of 20,737 proteins were quantified, but for differential accumulation across seasons we included only those quantified in both winter and summer samples (Table 1). Principal component analysis (PCA) of 745 orthogroups confirmed consistent clustering of replicates within species (Supplemental Figure S1A). For *Tripsacum* samples, we accounted for batch and year effects to evaluate potential variation among different hybrids. After controlling for these factors, seasonal variation emerged as the primary driver of protein expression differences among the 1,889 proteins analyzed, leading us to analyze all *Tripsacum* samples as a pooled species group (Supplemental Figure S1B).

### Cross-Species comparative proteomic analysis reveals conserved seasonal protein accumulation patterns

**Figure 2.**
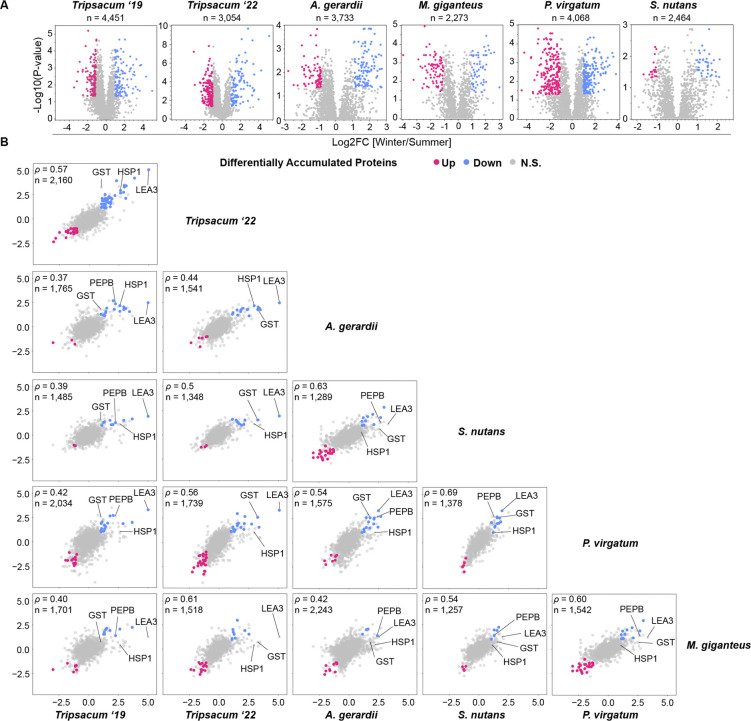
Proteomic analysis of rhizomes from PACMAD species under winter and summer conditions. **(A)** Volcano plots showing differentially accumulated proteins (DAPs) in rhizomes of *Tripsacum spp.*, *Andropogon gerardii*, *Miscanthus giganteus*, *Panicum virgatum*, and *Sorghastrum nutans* under winter vs. summer conditions, with total number of proteins identified listed. Proteins with significant upregulation (blue) and downregulation (pink) in winter relative to summer are highlighted, with non-significant proteins in gray. The y-axis represents the significance as −log_10_(p-value), and the x-axis shows the log_2_ fold-change (log_2_FC) of the DAPs between winter and summer. **(B)** Pairwise comparisons of log2 fold-change (Winter/Summer) in differentially accumulated orthogroups across species. Scatterplots display the correlation of orthogroup-level log2FC values between species, with shared DAPs upregulated in winter shown in blue, downregulated in red, and non-significant proteins in gray. Each panel represents a comparison between two species, with the number of shared orthogroups (*n*) and Spearman’s correlation coefficient (ρ) displayed.

Per species, the number of identified proteins ranged from 2,372 to 4,451. The number of differentially abundant proteins varied by species, with significantly upregulated proteins ranging from 58 to 174 and downregulated proteins from 56 to 218. Figure 2A shows that each species has a different number of proteins accumulated or depleted during winter relative to summer, suggesting that detection levels may be partly influenced by reference proteome completeness, as reflected by the comparatively lower coverage in *Sorghastrum nutans,* a tetraploid which has a 2Gb draft genome with a 15X coverage compared to 35–90X in the remaining species (Mitros et al. 2020; Stitzer et al. 2025).

To assess cross-species consistency at the orthogroup level, we identified shared protein families among the five species using OrthoFinder (Emms and Kelly 2019). Across all species, 51,442 orthogroups were identified in total (Supplemental Table S2). Of the 20,737 proteins quantified in this study, 18,454 were assigned to 4,888 orthogroups, while 2,283 lacked orthogroup assignment and were excluded from cross-species comparisons. The log_2_ fold-change values (winter vs. summer) of orthogroups were positively correlated across species, with specific orthogroups—such as LEA3, GST, HSP1, and PEPB, consistently identified as differentially abundant (Figure 2B). In cases where multiple proteins belonged to the same orthogroup within a species, log_2_ fold-change values were averaged to generate a single orthogroup-level fold change (Supplemental Table S3). Resulting Spearman correlations between orthogroup-level fold changes ranged from 0.37 (*Tripsacum* ‘19 vs. *A. gerardii*) to 0.69 (*S. nutans* vs. *P. virgatum*), with sample sizes ranging from 1,257 to 2,160 orthogroups. Comparisons within *Tripsacum* between 2019 and 2022 had a Spearman correlation of 0.56 (n = 2,160 orthogroups).

### Ortholog sharing

**Figure 3.**
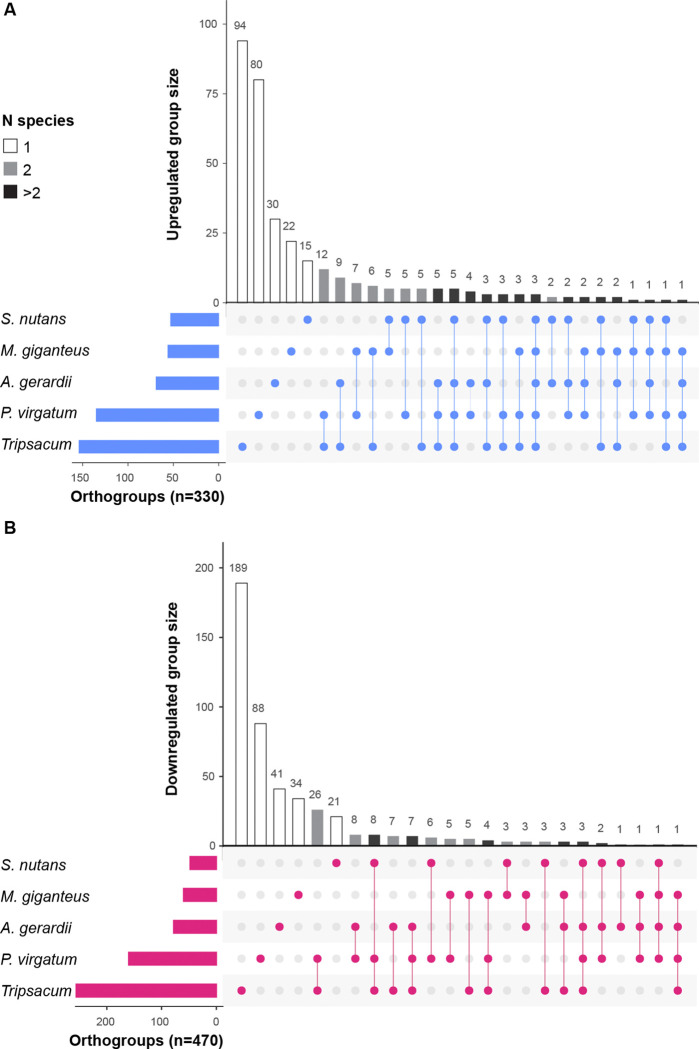
Orthogroup sharing of significantly DAPs among five PACMAD grass species. **(A)** Upregulated. (**B**) Downregulated. Set sizes (horizontal bars) indicate the total number of regulated orthogroups identified per species. Intersection sizes (vertical bars) represent orthogroups unique to individual species or shared among multiple species. Bar shading indicates the number of species sharing each orthogroup: unique to one species (white), shared between two species (gray), or shared among more than two species (black). Dots below the plot illustrate species-specific orthogroup intersections.

To evaluate the prediction that the same orthologs were shared, we compared differential accumulation of proteins by orthogroups (Figure 3 and Supplemental Tables S4 and S5). The biggest difference is caused by the initial proteome coverage (Table 1, Figure 2A) - *Tripsacum* and *Panicum* have the largest sets of unique proteins. Nevertheless, substantial sharing was observed across these independently evolved grass species. Of the total upregulated DAP orthogroups (n=330, Figure 3A), 87 proteins (approximately 27%) were shared with at least one other species, while 12% were shared among at least two additional species. In contrast, sharing of orthogroups among downregulated DAPs (n = 470, Figure 3B) was lower, with only 21% shared with at least one other species, and a mere 6% common to at least two additional species.

### Pathway sharing across species

While sharing was seen at the orthogroup level, we also tested if there was convergence at the pathway level. Functional categorization was carried out on 630 upregulated and 749 downregulated DAPs identified across species. Of these, 475 upregulated and 609 downregulated proteins had assigned GO terms and were used for enrichment analysis. This analysis highlighted clear distinctions between the two groups (Supplemental Figure S2A), with upregulated cold responses dominated by biological processes such as protein folding and stress chaperones, responses to heat and water deprivation, metabolic adjustments, detoxification and ROS scavenging, and signaling and membrane stabilization (Supplemental Table S4). In contrast, downregulated proteins primarily involved cytoskeletal and cell cycle processes, including microtubule-based dynamics, mitotic cell cycle progression, and nucleotide-sugar biosynthesis (Supplemental Table S5). Visual inspection of the 475 upregulated DAPs with GO annotations suggested shared response patterns across species, but a chi-square test indicated significant differences among PACMAD grasses (*p* = 0.012, Supplemental Figure S2B). However, this result may be misleading, as standard GO classifications are often too broad to accurately capture cold-specific biological processes (Barnes et al. 2023), prompting us to manually consolidate GO terms into more targeted cold-related categories (see Methods and Supplemental Figure S5).

**Figure 4.**
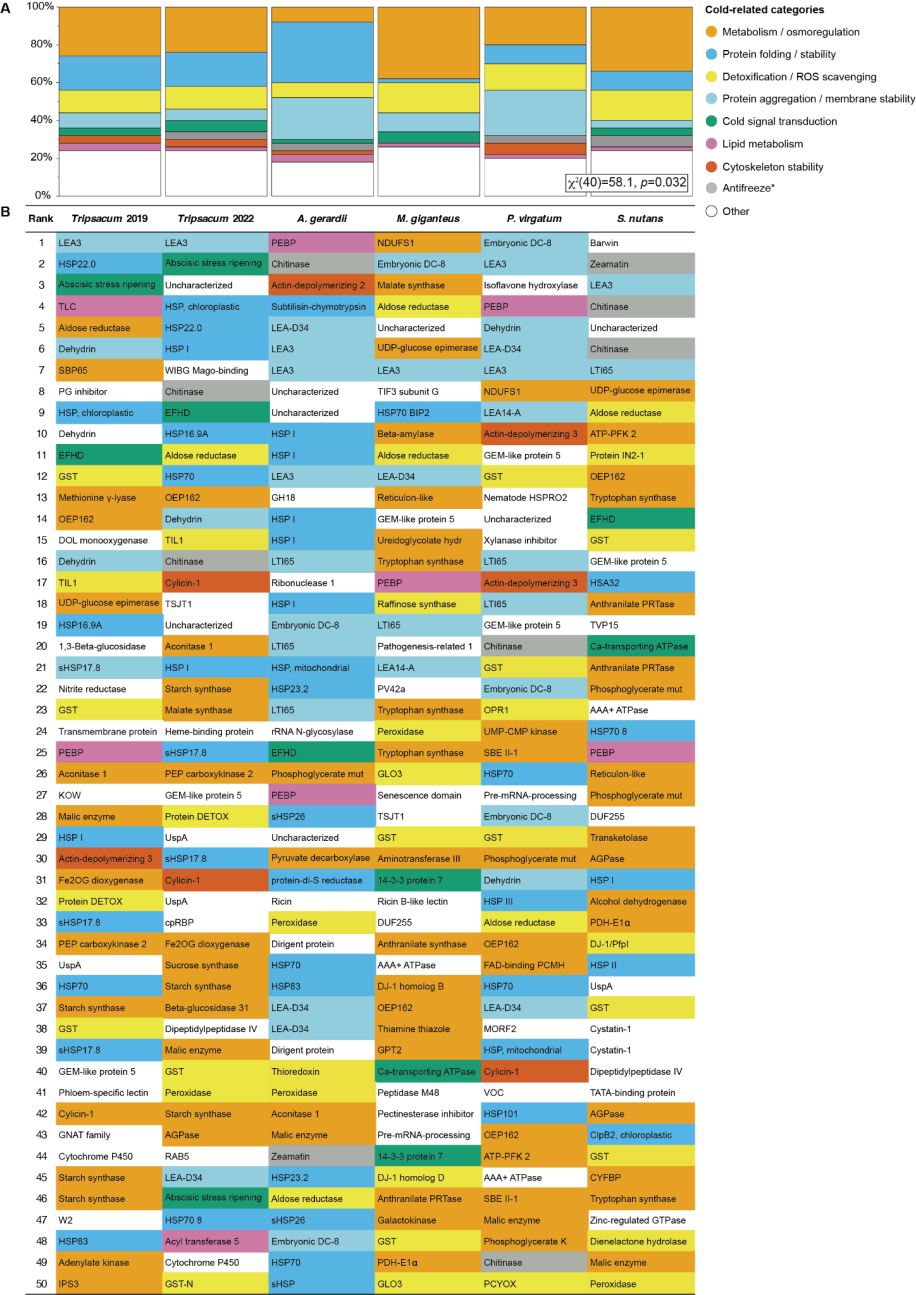
Overview of cold-related functional categories represented among the top 50 most highly upregulated rhizome proteins across five PACMAD grasses. **(A)** Mosaic plot of cold-related functional categories across species. This plot visualizes the distribution of proteins among cold-related functional categories for each species. The relative abundance of each category within a species is shown, highlighting distinct functional profiles and species-specific variations in cold tolerance mechanisms. A Chi-square test for independence (χ^2^[40] = 58.1, *p* = 0.032) was performed to evaluate variation across species. **(B)** Visual representation of cold-related functional categories in rhizome proteins of five Andropogoneae species. This figure illustrates the top 50 most abundant proteins within the rhizomes of five Andropogoneae species. Proteins are classified into functional categories relevant to cold-related mechanisms, such as metabolism/osmoregulation, cold signal transduction, membrane stability, lipid metabolism, and others. Color-coding facilitates a comparative visualization of cold tolerance mechanisms across species. Antifreeze proteins are marked by an asterisk, as their classification is based solely on protein homology.

To examine what pathways and functional protein classes were shared, we focused on the 50 most highly abundant proteins across each species (Figure 4, Supplemental Table S6). This revealed extensive sharing at the level of deeper homologs and pathways; for example, dehydrins, LEAs and HSPs were present in at least four of the five species. Using our approach to consolidate GO terms into specific cold-related categories, 63% of the proteins were binned into one of the nine categories (Supplemental Figure S5). Many of the remaining proteins are either unknown or may eventually be linked to these known processes with further investigation. Our analyses revealed both conserved functional cold-categories and species-specific variations in cold tolerance mechanisms (Figure 4A). Detoxification/ROS scavenging proteins were dominant across all species, highlighting a universal requirement for oxidative stress management. Proteins associated with primary metabolism and osmoregulation were abundant in most species, except *Andropogon gerardii*, indicating broad importance for energy balance and osmotic regulation. Proteins associated with protein aggregation and membrane stability proteins were particularly enriched in *A. gerardii* and *Panicum virgatum*. A chi-square test revealed a statistically significant variation in the distribution of cold-related functional categories across species (*p* = 0.03, Figure 4A). As shown in the figure, while all species upregulate the same core set of cold-related functional categories, their relative proportions differ significantly among species, particularly in metabolism/osmoregulation and protein folding/stability categories.

To identify specific proteins universally conserved across all five species, we examined the orthologs that are both abundant and consistently associated with cold tolerance (Figure 4B). LEA Protein Group 3 (LEA3, OG0022470) was the only ortholog detected in all five species, ranking among the most abundant proteins in each. Lipid-associated proteins were identified in multiple species. Phospholipid-binding proteins were present in all species. Other proteins classified within the top 10 included additional LEA proteins such as dehydrin, identified in specific species, as well as HSPs, including HSP22 in *Tripsacum* and the 23.2 kDa HSP in *Sorghastrum nutans*. Metabolic proteins were also highly ranked, with malate synthase among the most abundant in *Miscanthus giganteus* and aldose reductase present in *Tripsacum*, *Andropogon gerardii*, and *Panicum virgatum*. FAD-binding PCMH-type domain proteins were detected exclusively in *Panicum virgatum*. Signal transduction proteins were less frequently ranked in the top 10 but included a 39 kDa EF-hand containing protein common to *Tripsacum*, *Andropogon gerardii*, and *Sorghastrum nutans*. Putative antifreeze proteins such as chitinases and zeamatin were classified as such based on their sequence and structural homology to conifer chitinase and wheat thamatin respectively (Kontogiorgos et al. 2007; Jarząbek et al. 2009) ranked highly in *Sorghastrum nutans* and *A. gerardii* but were also present in *Tripsacum* and *P. virgatum*. ROS scavenging proteins were identified in all species, with glutathione transferase present in all except *Andropogon gerardii*, which exhibited peroxidases instead. Proteins associated with cytoskeletal components were present in multiple species, including actin-depolymerizing factors in *Andropogon gerardii* and *Panicum virgatum* (ranks 3 and 10, respectively), as well as in *Tripsacum*, and additional annotated components in *Miscanthus giganteus* and *Sorghastrum nutans*.

### Evolutionary patterns in upregulated protein families

**Figure 5.**
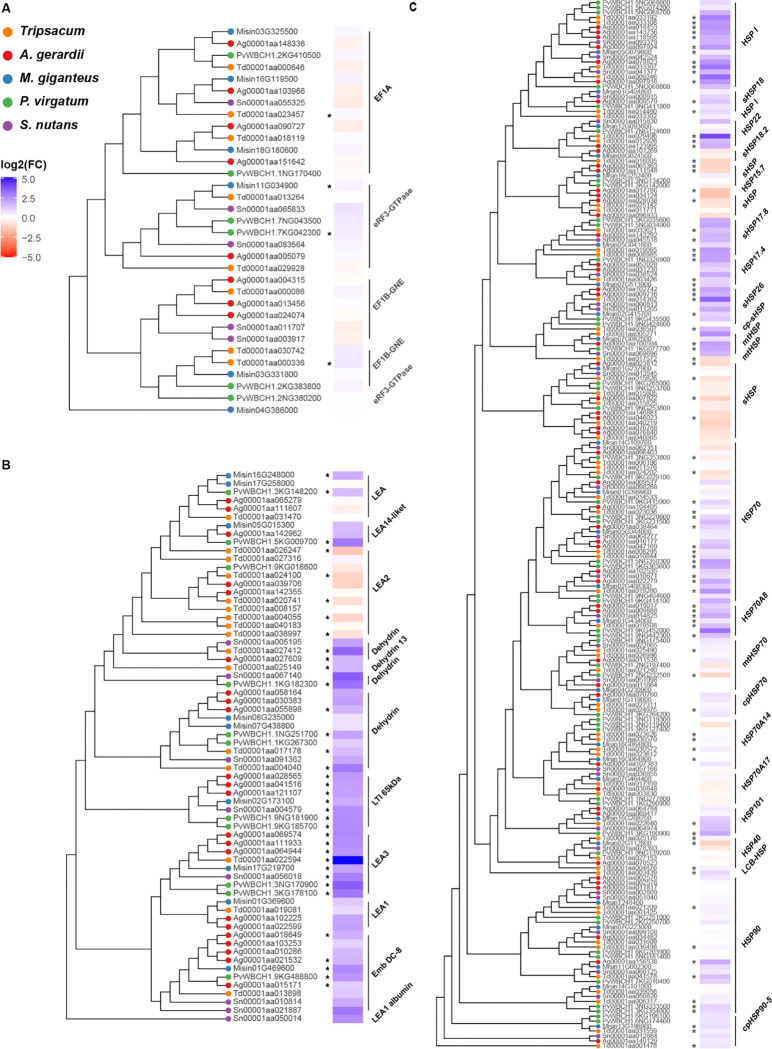
Phylogenetic analysis and seasonal abundance dynamics of selected protein families across five PACMAD grasses (*Tripsacum spp.*, *P. virgatum*, *A. gerardii*, *M. giganteus*, and *S. nutans*). Protein identifiers represent species-specific sequences, and brackets denote functional group annotations, merging adjacent orthogroups with similar functions for clarity. Heat maps illustrate log_2_ fold-change (log_2_FC) in protein abundance between winter and summer conditions, where blue indicates higher abundance in winter, and red signifies higher summer abundance. Panels show distinct protein families: **(A)** translation elongation factor 1 proteins (EF1; control group), **(B)** late embryogenesis abundant (LEA) proteins, and **(C)** heat shock proteins (HSPs). Asterisks (*) mark proteins exhibiting statistically significant differential accumulation (*p* < 0.05).

Phylogenetic analysis revealed distinct evolutionary patterns between late embryogenesis abundant (LEA) families and heat shock families (HSPs, Figure 5). The control elongation factor 1 protein (EF1) family, previously identified as among the most stably expressed genes across conditions (Takamori et al. 2017), Figure 5A) showed minimal seasonal regulation and no phylogenetic clustering of expression patterns, consistent with their housekeeping function (Supplemental Table S7). HSPs showed greater diversity (212 proteins across 37 orthogroups, Supplemental Table S9) compared to LEAs (66 proteins in 20 orthogroups, Supplemental Table S8), with control EF1 comprising 33 proteins in 4 orthogroups. Both LEA and HSP phylogeny (Figures 5B and C) resolved into clear functional sub-families, but in HSP the most strongly upregulated members (log_2_FC > 2.5) belonged to different clades across different species, with only 32% of HSP proteins significantly upregulated in winter. In contrast, LEA proteins (Figure 5B) demonstrated more consistent winter upregulation (47% of the proteins), particularly within the LEA3 subfamily, which displayed strong upregulation across all species despite having diverged earlier than other LEA clades.

### Beyond rhizomes: Comparison with maize & Tripsacum mRNA expression

Having identified season-long protein accumulation patterns conserved in rhizome cold tolerance across independently evolved PACMAD grasses, we next explored whether these signatures extended to the short lived mRNA in other tissues within *Tripsacum* and their orthologous genes in cold-sensitive maize. To compare rhizome protein accumulation to mRNA in maize and *Tripsacum* profiles, we first averaged *Tripsacum* protein fold-changes across both sampling years. These patterns were then compared with transcriptomic responses to cold in seedling leaves and other tissues. Cold-induced mRNA responses in F_2_ seedling leaves of *Tripsacum*, derived from the same hybrids used in our rhizome study, showed moderate correlation with rhizome protein levels (*ρ* = 0.29, Supplemental Figure S3A). Other *Tripsacum* tissues showed significant but weaker overlap (9–17 genes upregulated, Supplemental Figure S3B–E) as did maize (11–19 genes upregulated across tissues, Supplemental Figure S3F–I). LEA3 displayed strong differential expression consistently in leaves in both species.

### Structural and Functional Insights into LEA3: A Key Player in Freezing Tolerance

The only ortholog consistently found across all five studied species and highly ranked for its role in freezing tolerance, LEA3 (OG0022470) stands out as the top differentially accumulated protein (DAP) in Tripsacum dactyloides rhizomes with a 5-fold change in winter proteomics and as the most upregulated gene in leaves with an 8-fold change in mRNA expression. To explore its central role in cold adaptation and divergence in *Zea mays*, we examined the structural and functional features of LEA3, focusing on its sequence, motif conservation, and hydropathicity.

LEA3 proteins are defined by a repeating 11-mer motif, described by Dure as essential for desiccation and freezing tolerance (Dure III, 1993). Briefly, these motifs feature apolar (hydrophobic) residues at positions 1, 2, 5, and 9, forming the hydrophobic face of an α-helix critical for stabilizing cellular membranes and preventing protein aggregation. Threonine (T) can replace alanine (A) likely due to similar side-chain properties. Positions 6, 7, and 8 exhibit either a “+ - +” pattern (alternating positively and negatively charged residues) or a “+ Q +” arrangement.. LEA3 is known to form dimers or higher-order oligomers, likely stabilized by the alignment of conserved motifs and hydrophobic interactions.

**Figure 6.**
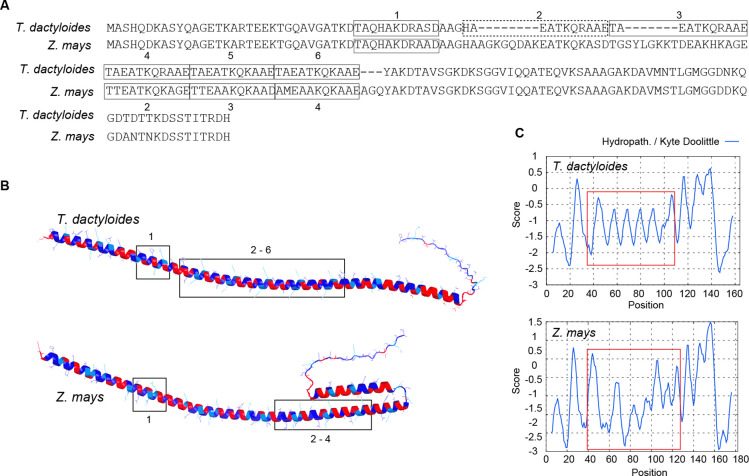
LEA3 protein sequence alignment, structure and hydrophobicity. **(A**) The alignment shows the LEA3 proteins from *T. dactyloides* (Td00001aa022594_T002) and, *Z. mays* (Zm00001eb294480), with the repeating 11-mer LEA3 motifs highlighted and labeled numerically. The dashed motif deviates from Dure’s LEA3 motifs in the first position. (**B**) Predicted secondary structure of LEA3 proteins, showing the conserved alpha-helical domains across *T. dactyloides* (top) and *Z. mays* (bottom). LEA motif regions corresponding to labeled sequence motifs (e.g., 1, 2–6) are boxed. Red and blue indicate regions of high hydrophilicity and hydrophobicity, respectively. **(C)** Kyte-Doolittle hydropathy plots for LEA3 proteins. The X-axis represents residue position, while the Y-axis indicates hydropathicity scores (positive values for hydrophobic regions, negative for hydrophilic regions). 11-mer motif regions are highlighted with red boxes, corresponding to key domains in (B).

*Tripsacum* LEA3 (Td00001aa022594_T002) sequence exhibits six consecutive 11-mer motifs that adhere closely to Dure’s rules. (Figure 6A). These motifs contribute to a well-structured, repeating motif pattern, supporting the formation of a stable hydrophobic face on the α-helix. These motifs are highly conserved and align with the α-helical domains identified in the structural mode (Figure 6B). The hydropathicity plot shows repetitive hydrophobic peaks corresponding to these motifs, reinforcing their importance in forming a robust hydrophobic interface (Figure 6C). In contrast, the alignment of *Z. mays* ortholog (Zm00001eb294480) contains four motifs and features a 15 amino acid insertion between consecutive motifs. This insertion disrupts regular motif spacing, as reflected in the structural model, and results in an irregular hydropathicity profile with breaks in hydrophobicity coinciding with the insertion (Figure 6C). Quantitative analysis of the 40–120 region reveals that Tripsacum LEA3 contains consecutive 11-mer motifs with consistent hydrophobicity at key positions (1, 2, 5, 9: −1.09 to −1.11), forming a continuous amphipathic pattern. In contrast, maize LEA3 shows significant disruption with more negative values at these same positions (−1.24 to −1.32), a 13–20% reduction in hydrophobicity. The greatest differences occur in positions 70–83 (ΔHydro = 0.72) and 87–101, where pattern correlation becomes strongly negative (*r* = −0.89), indicating a reversal of the hydrophobic/hydrophilic pattern. This disruption likely impairs the formation of amphipathic helices necessary for membrane stabilization during freezing (Supplemental Table S10). Hydropathy plots for other PACMAD grasses, as well as closest orthologues in *T. aestivum* (TdLEA3; (Koubaa et al. 2019) and *A. thaliana* (AtLEA7; (Popova et al. 2015), reveal conserved oscillating patterns similar to *Tripsacum*, further highlighting the divergence of the *Z. mays* LEA3 profile (Supplemental Figure S4).

## Discussion

Despite their diverse evolutionary histories and origins in warmer climates, PACMAD grasses have convergently adapted to cold environments, offering a unique opportunity to study the genetic and proteomic basis of cold resilience in warm-adapted lineages. Our proteomic analysis across five PACMAD grasses reveals strong evidence of conserved cold adaptation mechanisms despite distinct evolutionary trajectories spanning Gondwanan lineages (e.g., *Panicum virgatum*) to Southeast Asian radiations (e.g., *Miscanthus giganteus,* (Schubert et al. 2020; Gallaher et al. 2022). Central among these responses is the significant upregulation of proteins involved in detoxification and reactive oxygen species (ROS) scavenging, metabolism and osmoregulation, protein folding and stability, and membrane stabilization – processes universally essential to maintaining cellular homeostasis and structural integrity during cold stress (Suzuki and Mittler 2006; Gill and Tuteja 2010)

Co-occurrence analysis of cold-responsive orthogroups across species (Figure 3) revealed significant commonality in upregulated proteins across species, while downregulated proteins showed more species-specific patterns. This suggests that cold adaptation in PACMAD grasses likely occurred through conserved or convergent mechanisms underscoring the need for future studies to establish whether these were independent gains of function, or shared ancestral traits. In contrast, species-specific downregulation reflects unique developmental and metabolic adjustments to dormancy. This pattern is consistent with the concept that stress tolerance often evolves through conserved pathways, but the exact implementation may vary across evolutionary lineages (Bohnert et al. 1995; Schubert et al. 2019)

Because conventional GO terms lack specificity for cold adaptation (Barnes et al. 2023), we grouped proteins into focused cold-related categories, revealing conserved stress-responsive processes and highlighting the need for more tailored annotations in future omics-based stress studies (Figure 4A, Supplemental Figure S5). Unlike previous studies focusing predominantly on membrane lipid remodeling as central adaptations (Barnes et al. 2016; Surber et al. 2024), our findings highlight the critical roles of intrinsically disordered proteins, particularly late embryogenesis abundant (LEA) proteins. The consistent presence of LEA3 (OG0022470) as the only protein upregulated across all five independently evolved lineages suggests its fundamental importance in cold adaptation. This conservation indicates strong purifying selection, likely due to LEA3’s essential role in freezing tolerance. The early divergence from other LEA proteins (Figure 5B) suggests that the molecular properties enabling its cryoprotective function may have evolved before the radiation of PACMAD grasses. While ancestral PACMAD lineages likely did not experience freezing conditions (Gallaher et al. 2022), these properties may have originally served other stress-related functions and were later co-opted for freezing tolerance in temperate-adapted lineages (Schat et al. 2025).

The phylogenetic distribution of seasonally regulated proteins (Figure 5, Supplemental Tables S7–S9) provides important evolutionary insights beyond those offered by enrichment analyses alone. The greater diversity and larger size of the HSP family compared to LEAs likely reflects broader roles in proteostasis beyond stress responses (Wang et al., 2004). Observed upregulation patterns among HSPs suggest conserved chaperone functions during winter adaptation, although specific proteins recruited for this role appear to have diverged during PACMAD grass evolution. For example, Heat shock 70 kDa protein 8 (OG0021022) and Heat shock protein 70 (OG0000034) show conserved but quantitatively variable upregulation across species (Figure 5C, Supplemental Table S9), similar to patterns of regulatory subfunctionalization among HSP70s reported in *Eucalyptus* species (Ahrens et al. 2022). Species-specific signatures are also evident: Tripsacum strongly upregulates Small heat shock protein 26 (OG0016578) and Class IV heat shock protein (OG0016115), while A. gerardii and P. virgatum predominantly upregulate mitochondrial Heat shock protein (OG0015134), consistent with findings in *Brachionus rotifers*, where closely related species differentially recruited distinct HSPs in response to thermal stress (Paraskevopoulou et al. 2020). These patterns of both conserved and species-specific HSP upregulation suggest distinct paralog recruitment across lineages and support the idea of independent regulatory subfunctionalization within this family. Thus, regulatory evolution appears central to HSP adaptation to cold stress.

Cold-responsive proteins in *Tripsacum* rhizomes correlate significantly with mRNA responses across its tissues, strongest in leaves (Supplemental Figure S3). In maize, correlations are weaker or absent. LEA3 consistently shows strong differential expression across *Tripsacum* tissues but reduced responsiveness in maize, potentially contributing to species differences in cold tolerance.

The structural and functional analysis of LEA3 (Figure 6) provides mechanistic insights into cold tolerance differences between *Tripsacum* and maize. LEA proteins function as molecular shields during freezing stress through multiple mechanisms including membrane stabilization, ion sequestration, and chaperone activity (Kovacs et al. 2008; Cuevas-Velazquez et al. 2017). The unique 15-amino-acid insertion in *ZmLEA3* likely disrupts the regular spatial arrangement of conserved 11-mer motifs described by (Dure 1993). Changes in hydrophobic balance (Figure 6C) or steric hindrance may further limit the ability of *ZmLEA3* to bind membranes and stabilize cellular structures. The repeating hydropathy oscillation seen in most LEA3 proteins, including Arabidopsis and *Triticum aestivum* suggests a conserved structural motif in lineages with strong cold or desiccation tolerance that takes advantage of alternating hydrophobic residues to support folding into amphipathic α-helice (Supplemental figure S4). This pattern is thought to facilitate reversible binding to partially unfolded proteins or membrane surfaces during dehydration and freezing (Koubaa et al. 2019; Knox-Brown et al. 2020; Singh and Graether 2021). In contrast, *ZmLEA3* lacks this periodicity, potentially compromising its structural plasticity and interaction capacity, such changes likely influence LEA3 partner preferences or binding dynamics, leading to functional divergence.

Additional conserved cold-tolerance mechanisms identified in our study include antifreeze proteins (e.g., zeamatin, chitinases), crucial for mitigating ice damage (Yeh et al. 2000; Kontogiorgos et al. 2007; Jarząbek et al. 2009), and phospholipid-binding proteins that enhance membrane stability (Degenkolbe et al. 2012). The widespread presence of HSPs, notably HSP22 and the 23.2 kDa HSP, underscores the general requirement for stabilizing protein structure and function under freezing conditions (Thomashow 1999). Metabolic proteins such as malate synthase and aldose reductase facilitate energy provision and osmotic regulation during dormancy, reflecting adaptations to unique ecological pressures (Lee and Chen 1993).

Our study also reveals interesting connections between drought and cold tolerance mechanisms, as evidenced by the significant presence of ABA-inducible proteins and dehydrins in cold-upregulated proteins. This overlap suggests that cold tolerance in PACMAD grasses may have evolved partly by co-opting existing drought tolerance pathways (Schat et al. 2025), a common evolutionary strategy where new adaptive traits build upon existing stress response networks.

In conclusion, our comprehensive study demonstrates that the independent evolution of rhizome frost tolerance in PACMAD grasses appears to be governed by similar mechanisms driven primarily by expression-level changes and complemented by protein structural adaptations, particularly within the LEA family. Despite their diverse evolutionary histories and biogeographic origins spanning multiple continents, these grasses have remarkably similar proteomic solutions to cold and freezing stress. LEA3 stands out as the only protein consistently upregulated across all five species, highlighting its fundamental role in this convergent adaptation. The structural differences we identified between *Tripsacum* and maize LEA3 proteins offer promising targets for enhancing freezing resilience in cold-sensitive crops like maize. Future work should focus on functional validation of these key proteins to translate these evolutionary insights into practical breeding applications that could extend growing seasons in temperate regions.

## Materials and Methods

To investigate proteomic responses to seasonal changes across prairie grasses, we conducted a comparative analysis of rhizomes from five species under winter (January, with lows reaching −29°C) and summer (August, with highs of 26°C) conditions. The species analyzed were *Tripsacum*, *Andropogon gerardii*, *Miscanthus giganteus*, *Panicum virgatum*, and *Sorghastrum nutans* (Figure 1A).

### Plant material

Plants were grown outdoors in Ithaca, New York (USDA Hardiness Zone 6a), where they experienced natural environmental fluctuations, including deep-freezing winters. Tripsacum rhizomes were sampled from seven-year-old hybrid progenies at Cornell University’s Caldwell Field, representing crosses between northern (Iowa, Kansas, Illinois, Missouri) and southern (Florida, Texas) founders. These included a broader panel of accessions compared to the other species, and samples were collected in both 2019 and 2022 (see Table 1 for details). The other four species were represented by three accessions each, grown at the Cornell Botanical Gardens in parallel field plots.

F_2_ seeds used for seedling freezing tolerance screening and RNA sequencing were generated from the same *Tripsacum* hybrids described above. Due to the limited seed set achieved through self-pollination (~500 seeds over the entire season), F₁ plants were allowed to open-pollinate. Seeds were harvested from individual maternal plants, germinated in trays, and grouped into F_2_ families based on maternal lineage. Six-week-old F_2_ seedlings sharing the same maternal F₁ parent were selected as experimental material for freezing assays and RNA extraction.

### Proteomics data

Proteomic profiling was conducted using tandem mass tag (TMTpro) labeling and Orbitrap Eclipse Real Time Search (RTS)-SPS-MS3 technology. The experimental design included samples collected in both winter (January) and summer (August), with each species represented by at least three biological replicates with a larger number of genotypes analyzed for *Tripsacum* across two years (see Table 1 and Figure 1D).

### Protein Extraction and Digestion

Fresh SDX was ground in liquid nitrogen. The ground sample was suspended in a lysis buffer, composed of 1× PBS (pH 8), 10 mM EDTA, 900 mM sucrose, and 0.4% β-mercaptoethanol. The suspension was vortexed and incubated on ice, then mixed with an equal volume of phenol buffered to pH 8 with Tris-EDTA (TE). After centrifugation and phase separation, the phenol phase containing proteins was collected. Proteins were precipitated from the phenol phase using ice-cold ammonium acetate /methanol solution (100 mM w/v ammonium acetate in 100% methanol) with protease and phosphatase inhibitors. After precipitation the pellets were washed 5 times with 3 different solutions and frozen. Then protein pellets were dissolved in 8-M urea buffer. Protein concentration was determined using a BCA protein assay. A set amount of each protein sample underwent S-Trap digestion, including treatment with dithiothreitol and iodoacetamide. The sample underwent multiple rounds of centrifugation with the addition of 8-M urea, followed by 50-mM ammonium bicarbonate (ABC) or triethylammonium bicarbonate (TEAB). The sample was digested using a trypsin buffer, centrifuged, and then dried by vacuum freeze-drying for storage prior to subsequent TMT labeling.

### TMT Labeling and LC–MS/MS Analysis

Digested peptide mixtures were labeled using 10-plex TMT reagent, then pre fractionated using an Ultimate 3000 instrument. Samples were injected into nanoLC tandem Orbitrap Eclipse by a RTS-SPS-MS^3^ method (Fu et al. 2021). Raw MS data were processed in Proteome Discoverer 2.4 using the Sequest HT search engine. Spectra were searched against the UniProtKB database supplemented with common contaminants. Searches allowed up to two missed cleavages and included fixed modifications of TMTpro tags on peptide N-termini and lysine residues, and carbamidomethylation of cysteine. Dynamic modifications included methionine oxidation and asparagine/glutamine deamidation. Precursor and fragment mass tolerances were set to 10 ppm and 0.02 Da, respectively. Peptide spectral matches were filtered using Percolator with a 1% false discovery rate (FDR). Protein quantification was based on the summed reporter ion intensities of unique and razor peptides, filtered with a minimum signal-to-noise ratio of 10 and a co-isolation threshold of 50%. Total peptide amount normalization was applied across TMT channels. Differentially abundant proteins (DAPs) were determined based on a |log_2_ fold change| ≥ 1 cutoff and adjusted p-value < 0.05.

### Proteomics Fractionation

10 fractions used for MS/MS were pools of individual wells from high pH first-dimension fractionation. The high pH fractionation method collected 48 fractions in a standard 96-well plate. The “meandering scheme” collected fractions row by row. The first fraction was collected in well A1, the 13th fraction in well B12, continuing until D1. A second fractionation could use wells E1 to H1. Using a 2.1 × 150mm column at 200μL/min, the first well (assuming the first 3 wells A1-A3 were rejected) combined wells A4 + B11 + B1 + C10 + D5 for LC-MS/MS fraction 1. Pool 2 combined wells A5 + B10 + C1 + C11 + D4 for fraction 2, continuing until all useful wells were included in the 10 fractions for LC-MS/MS.

### Peptide Mapping and Reference Proteome Assignment

Due to multiplexing constraints, not all species were initially searched against species-specific reference proteomes. For species with <15% predicted proteome divergence, such as *Andropogon gerardii* and *Miscanthus giganteus*, samples were pooled in the same TMT batch and searched against the *A. gerardii* proteome. Inspection of peptide-level assignments revealed that numerous *M. giganteus* peptides mapped to *A. gerardii* proteins lacking clear orthologous relationships, resulting in reduced protein recovery for *M. giganteus.* To address this, *M. giganteus* peptides were re-mapped to the *M. sinensis* proteome—the closest available high-quality reference for *M. giganteus*—using a targeted R pipeline (see Supplemental Code S1–S3; GitHub repository). Briefly, TMT channels were matched to sample metadata, and peptides from the *M. giganteus* samples were re-aligned to the *M. sinensis* proteome using DIAMOND BLASTp in “ultrasensitive” mode. Subsequently, Proteins originally assigned using the *A. gerardii* reference proteome were reassigned to their best-matching *M. sinensis* counterparts based on aggregated peptide evidence. This was done using a tiered tie-breaking scheme that prioritized (1) the number of distinct peptides mapped, (2) the sum of DIAMOND bitscores, and (3) the median percent identity. Protein-level summaries were regenerated using these updated associations while preserving the original normalized peptide intensities, improving species-specific assignment without altering quantification.

All final analyses were conducted using species-specific reference proteomes: *Andropogon gerardii* (Ag-CAM1351-DRAFT-PanAnd-1.0), *Miscanthus giganteus* (Msinensis_497_v7.0), Panicum virgatum *(Pvirgatumvar_WBCHAP1_778_v1.0),* Sorghastrum nutans (Sn-CAM1369-DRAFT-PanAnd-1.0), and *Tripsacum dactyloides* (Td-FL_9056069_6-DRAFT-PanAnd-1.0); genome assemblies and annotations are listed in the Data Availability section.

### Protein abundance normalization and statistical analysis

Protein intensities were normalized using the “total peptide amount” method in Proteome Discoverer 2.4, ensuring comparability across TMT channels. Differential protein abundance between winter and summer was assessed using one-way ANOVA, followed by Tukey’s Honest Significant Difference post hoc test followed by multiple testing correction using the Benjamini-Hochberg procedure. Quantitative values were log_2_-transformed and proteins were considered differentially accumulated if |log_2_ fold change| ≥ 1 and adjusted p-value < 0.05. All statistical analyses and visualization were performed in R version 4.4.3, using RStudio 2024.12.1+563 (“Kousa Dogwood” release).

### Functional annotation and GO enrichment

Orthogroups were assigned using OrthoFinder (v2.5.5, Emms and Kelly 2019), clustering protein sequences from all five species based on sequence similarity and inferred gene trees. DIAMOND (v2.1.9, Buchfink et al. 2021) was used for all-vs-all sequence comparisons. Orthogroups served as the basis for cross-species comparisons and functional propagation of annotations, particularly for proteins with limited annotation coverage outside *Tripsacum dactyloides.*

Functional annotation was performed separately for DAPs and non-significant proteins. DAP sequences were first annotated using DIAMOND BLASTp against the SwissProt database. Protein descriptions and Gene Ontology (GO) terms were extracted from UniProt using a custom script. For DAPs lacking SwissProt hits, functional predictions were generated using PANNZER2 (Törönen and Holm 2022), run independently for each species. In contrast, for non-significant proteins, functional annotations were propagated from *Tripsacum dactyloides* to orthologous proteins within the same orthogroup when available. Remaining unannotated entries were manually curated based on sequence similarity and conserved domain features.

GO term enrichment analysis was performed using the topGO R package (v2.50.0), applying the “elim” algorithm with Fisher’s exact test to reduce redundancy and account for GO hierarchy. The background set included all proteins quantified across the experiment. Enrichment was conducted separately for proteins upregulated and downregulated in winter. Terms with a minimum node size of 10 and a p-value < 0.01 were considered significant. The top 15 enriched Biological Process (BP) terms in each group were visualized using dot plots in ggplot2, incorporating log-transformed p-values, gene ratios, and counts of significant proteins.

### Candidate protein exploration methods

(Barnes et al. 2023) showed that cold-responsive transcriptional and proteomic changes often overlap with broader stress mechanisms involving damage, osmotic imbalance, and acidification. To improve biological resolution, we curated DAPs into nine functional categories based on known roles in freezing tolerance (Örvar et al. 2000; Iwaya-Inoue et al. 2018; Schubert et al. 2020) : (1) protein aggregation and membrane stability, (2) protein folding and chaperone activity, (3) metabolism and osmoregulation, (4) detoxification and ROS scavenging, (5) lipid metabolism, (6) antifreeze and cell wall modification, (7) cold signal transduction, (8) cytoskeletal organization, and (9) other/uncategorized. Assignments were informed by protein descriptions, GO terms, PANNZER2 predictions, and manual review of domain features and relevant literature. This categorical framework enabled a clearer visualization of conserved and divergent cold responses across species (Supplemental Figure S5), particularly by consolidating functionally relevant proteins that may be dispersed across non-specific GO categories.

### Selecting proteins for traits on trees

Proteins for phylogenetic trait mapping were identified by text‐mining functional annotations produced by PANNZER2 for both the *Tripsacum dactyloides* proteome and the DAP sets from all five species, searching specifically for GO terms associated with late embryogenesis abundant (LEA) proteins, heat shock proteins (HSPs), and elongation factor 1 (EF1). Extracted orthogroup identifiers from our log_2_ fold‐change matrix were then used to retrieve the corresponding protein IDs, which served as input for sequence extraction. Full‐length amino‐acid sequences were obtained from each species’ reference proteome using seqkit grep (v2.0) with protein headers matching these IDs. Multiple sequence alignments were generated in MAFFT (v7.475) under default settings, and maximum‐likelihood and maximum‑likelihood trees were inferred using raxmlHPC (RAxML‑8.2.13) PROTGAMMAJTT model with 100 rapid bootstraps. Each resulting tree was rooted on a biologically appropriate outgroup (e.g., a chloroplastic class I small HSP) and visualized in R (v4.1.0) using the ggtree package, with log_2_FC values mapped as a color gradient onto tip points.

### RNA-seq processing and differential expression analysis

Transcriptome data for maize chilling responses across tissues (leaf, root, top-crown, and bottom-crown) were derived from (Xue et al. 2021), which profiled transcriptomic responses to chilling stress in B73 using 3′ RNA-seq (5C vs. 22C). For *Tripsacum*, we analyzed two datasets. The first comprised leaf transcriptome data obtained from a segregating freezing-tolerant bulk of F_2_ seedlings derived from the same Tripsacum hybrids used in our rhizome analysis. Plants from seven F_2_ families were bulked as freezing-tolerant or intolerant based on survival after exposure to −8°C for three hours. Within the freezing-tolerant bulk only, RNA was collected from 6-week-old seedlings at control temperature (22°C) and after 7 days of cold acclimation (5°C), with expression compared between these conditions (tol_5C vs. tol_22C). The second dataset included transcriptome data from additional *Tripsacum* tissues (leaf, root, top-crown, and bottom-crown) generated from *Tripsacum* ‘Pete’ using the same methodology and comparison (5C vs. 22C) as in (Xue et al. 2021) and are currently unpublished.

F_2_ seedling leaf RNA was extracted using the methods described in (Gault et al. 2018). Paired-end RNA-seq reads were quality-filtered and trimmed using Trimmomatic (version 0.32, Bolger et al. 2014) to remove Illumina adapter bases discarding bases from beginning and end with a phred score <35 or <20, respectively. Reads with a minimum length of 36 bp were retained. Reads were aligned to the *Tripsacum dactyloides* genome (Td-FL_9056069_6-DRAFT-PanAnd-1.0.fasta) and annotation (Td-FL_9056069_6-DRAFT-PanAnd-1.0_final.gff3) using STAR v2.7.10b (--twopassMode Basic), allowing each read to map to up to 20 locations (--outFilterMultimapNmax 20). Gene-level counts were extracted from uniquely mapped reads using featureCounts v2.0.3 with default settings. Raw counts were normalized using the estimateSizeFactors method in DESeq2, which adjusts for sequencing depth and RNA composition bias. Differential gene expression analysis was conducted using DESeq2 (Love et al. 2014). Contrasts were made between 7 °C and 22 °C within each genotype (eight F_2_ families) with 5 °C as the reference, modeling both temperature and genotype as main effects (~ genotype + temperature). Genes were considered differentially expressed if they passed an adjusted p-value cutoff of 0.05 (Benjamini-Hochberg correction) and showed a minimum absolute log_2_ fold change > 1.

### Structural and Biophysical Analysis of LEA3 Proteins

Amino acid sequences of LEA3 orthologs from *Tripsacum dactyloides* (Td00001aa022594_T002) and *Zea mays* (Zm00001eb294480) were aligned using MAFFT v7.475 with default settings. Predicted three-dimensional structures were generated using AlphaFold3. Hydrophobicity was visualized in PyMOL (v2.5) using a custom script based on the Eisenberg scale, which colored residues according to side-chain properties. Hydropathy plots were generated using both ProtScale (ExPASy) and AmphipaSeek (Sapay et al. 2006), with a sliding window size of 11 amino acids corresponding to the LEA3 motif length. Hydrophobic peaks associated with conserved LEA motifs were annotated across both sequence alignments and structural models.

## Supplementary Material

Supplement 1 **Supplemental Figure S5**. Stacked bar chart illustrating the composition of BPs within curated cold-related functional categories. The plot includes 230 proteins from the top 50 DAPs upregulated in winter across species, after excluding those assigned to the cold response category “Other” (i.e., proteins with annotations deemed less relevant to cold tolerance interpretation). Cold-related categories are shown on the x-axis and sorted by total protein count. Each bar is color-coded by BP, highlighting the functional composition of each category. Less informative or poorly annotated BPs were grouped under “Other” in the legend. This visualization supports the manual consolidation of specific biological processes into broader cold-response categories used throughout the study.

Supplement 2

## Figures and Tables

**Table 1.** Summary of rhizome proteomics datasets, including species, accession, year, number of samples per season, proteins quantified, proteins shared across seasons, and differentially accumulated proteins (DAPs) up- or downregulated in winter. Total protein counts before reproducibility filtering are shown in parentheses for Tripsacum hybrids.

Species	Accession	Year	N samples	Proteins quantified		Sig Up	Sig down
				Total	Shared Winter/Summer		

*Tripsacum spp.*	*T. dactyloides* ‘KS’ × *T. floridanum* *T. dactyloides* ‘IA’ × *T. dactyloides* ‘FL’ *T. dactyloides* ‘MO’ × *T. dactyloides* ‘TX’	2019	8	4,451	4,451	123	175
*Tripsacum spp.*	*T. dactyloides* ‘KS’ × *T. floridanum* *T. dactyloides* ‘IL’ × *T. floridanum* *T. dactyloides* ‘IL’ × *T. dactyloides* ‘FL’ *T. dactyloides* ‘IA’ × *T. dactyloides* ‘FL’ *T. dactyloides* ‘MO’ × *T. dactyloides* ‘TX’	2022	24	3,468	3,054	100	141
*A. gerardii*	‘Sentinel’	2022	3	3,734	3,733	109	76
*M. giganteus*		2022	3	2,372	2,273	58	83
*P. virgatum*	‘Dust Devil’	2022	3	4,071	4,068	174	218
*S. nutans*	‘Golden Sunset’	2022	2	2,641	2,464	66	56

## Data Availability

The mass spectrometry proteomics data have been deposited to the ProteomeXchange Consortium via the PRIDE (Perez-Riverol et al. 2025) partner repository with the dataset identifier PXD063668. RNA-seq data are available under BioProject accession numbers PRJNA705456 (*Zea mays*) and PRJNA1260937 (*Tripsacum dactyloides*) in the NCBI BioProject database (https://www.ncbi.nlm.nih.gov/bioproject/). Genome assemblies and annotations used in this study are publicly available from MaizeGDB (Woodhouse et al. 2021) at https://maizegdb.org/PanAnd_project for *Tripsacum dactyloides* (Td-FL_9056069_6-DRAFT-PanAnd-1.0), *Andropogon gerardii* (Ag-CAM1351-DRAFT-PanAnd-1.0), and *Sorghastrum nutans* (Sn-CAM1369-DRAFT-PanAnd-1.0), and from the JGI Data Portal (Goodstein et al. 2012) at https://data.jgi.doe.gov/ for *Miscanthus giganteus* (Msinensis_497_v7.0) and *Panicum virgatum* (Pvirgatumvar_WBCHAP1_778_v1.0), along with their corresponding GFF annotation files. Functional annotations, data tables, and analysis scripts are available from GitHub at https://github.com/elor77/PACMAD-Rhizome-Proteomics.
