# Supplementary material for: Grass Rhizome Proteomics Reveals Convergent Freezing-Tolerance Strategies
Source: bioRxiv. 2025 May 19:2025.05.15.654294. Preprint. [Version 1] doi: 10.1101/2025.05.15.654294 (PMC12258727; doi:10.1101/2025.05.15.654294)
Supplement: Supplement 1 — Supplemental Figure S5. Stacked bar chart illustrating the composition of BPs within curated cold-related functional categories. The plot includes 230 proteins from the top 50 DAPs upregulated in winter across species, after excluding those assigned to the cold response category “Other” (i.e., proteins with annotations deemed less relevant to cold tolerance interpretation). Cold-related categories are shown on the x-axis and sorted by total protein count. Each bar is color-coded by BP, highlighting the functional composition of each category. Less informative or poorly annotated BPs were grouped under “Other” in the legend. This visualization supports the manual consolidation of specific biological processes into broader cold-response categories used throughout the study. [file media-1.pdf]

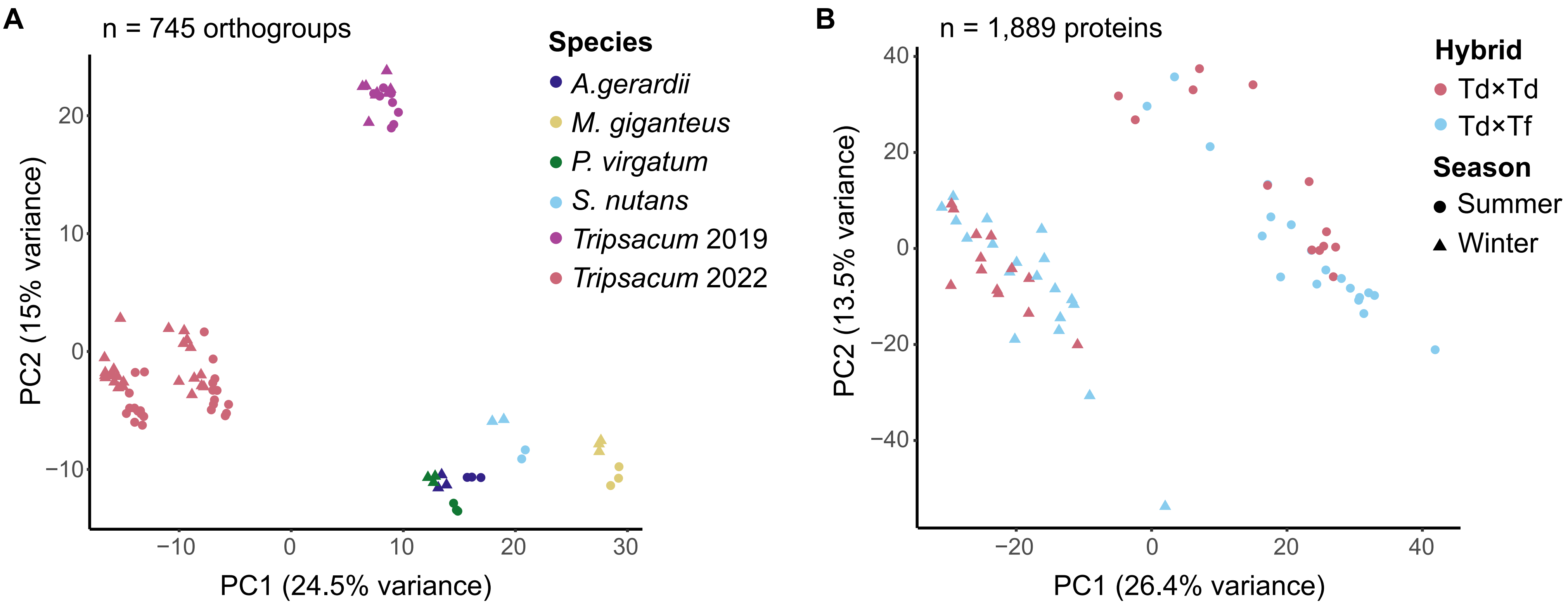

Supplemental Figure S1. Principal component analysis (PCA) of log-transformed protein abundance data. (A) PCA of 745 orthogroups from five species *Tripsacum* (Td-2019, Td-2022), *A. gerardii* (Ag), *M. giganteus* (Mg), *P. virgatum* (Pv), and *S. nutans* (Sn). **(B)** PCA of 1,889 proteins from *Tripsacum* hybrids. Points are colored by hybrid type–intraspecific *T. dactyloides* hybrids (Td × Td) and interspecific *T. dactyloides* × *T. floridanum* hybrids (Td × Tf). Shapes represent season (circle = summer, triangle = winter). The percentage of total variance explained by each principal component is shown on the axes.

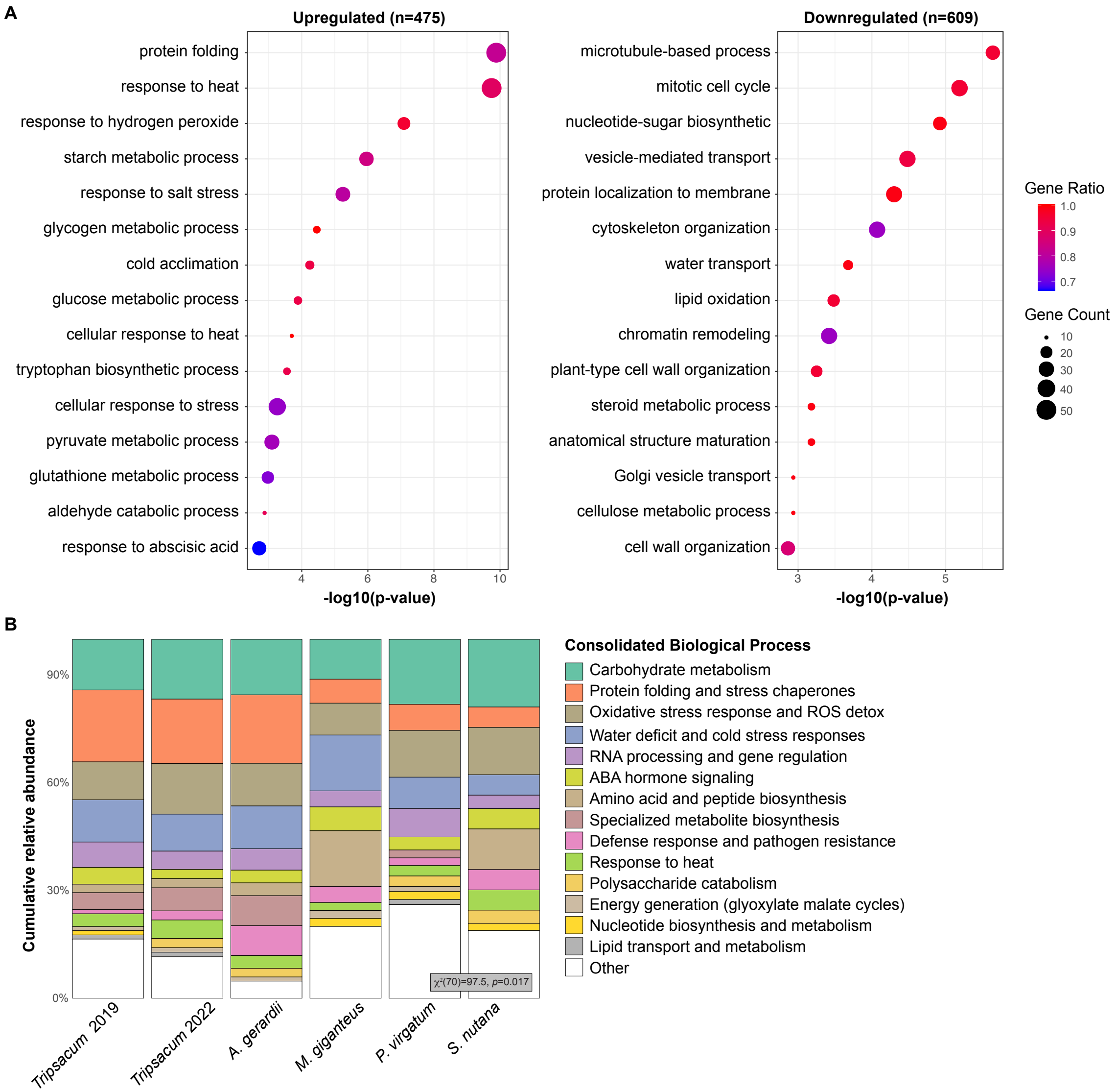

Supplemental Figure S2. (A) Enriched biological processes (BP) among differentially accumulated proteins (DAPs) in 475 upregulated and 609 downregulated proteins (proteins with unknown BP term were excluded). Dot size represents gene count per BP category, while dot color indicates the gene ratio (proportion of proteins annotated to each BP category). Only the top significantly enriched BP terms (lowest p-values) are displayed for clarity. **(B)** Mosaic plot of biological processes enriched among upregulated DAPs across five Andropogoneae species. The plot summarizes the distribution of functionally annotated proteins (n = 483 of 630; unannotated proteins were excluded) across 139 biological process (BP) terms. These terms were consolidated into broader functional categories to aid interpretability. Each bar shows the relative abundance of functional categories within a species, highlighting both conserved and species-specific patterns. A Chi-square test for independence ($\chi^2[70]=97.5$, $p=0.017$) was performed to evaluate variation across species. Category labels are shown in the legend.

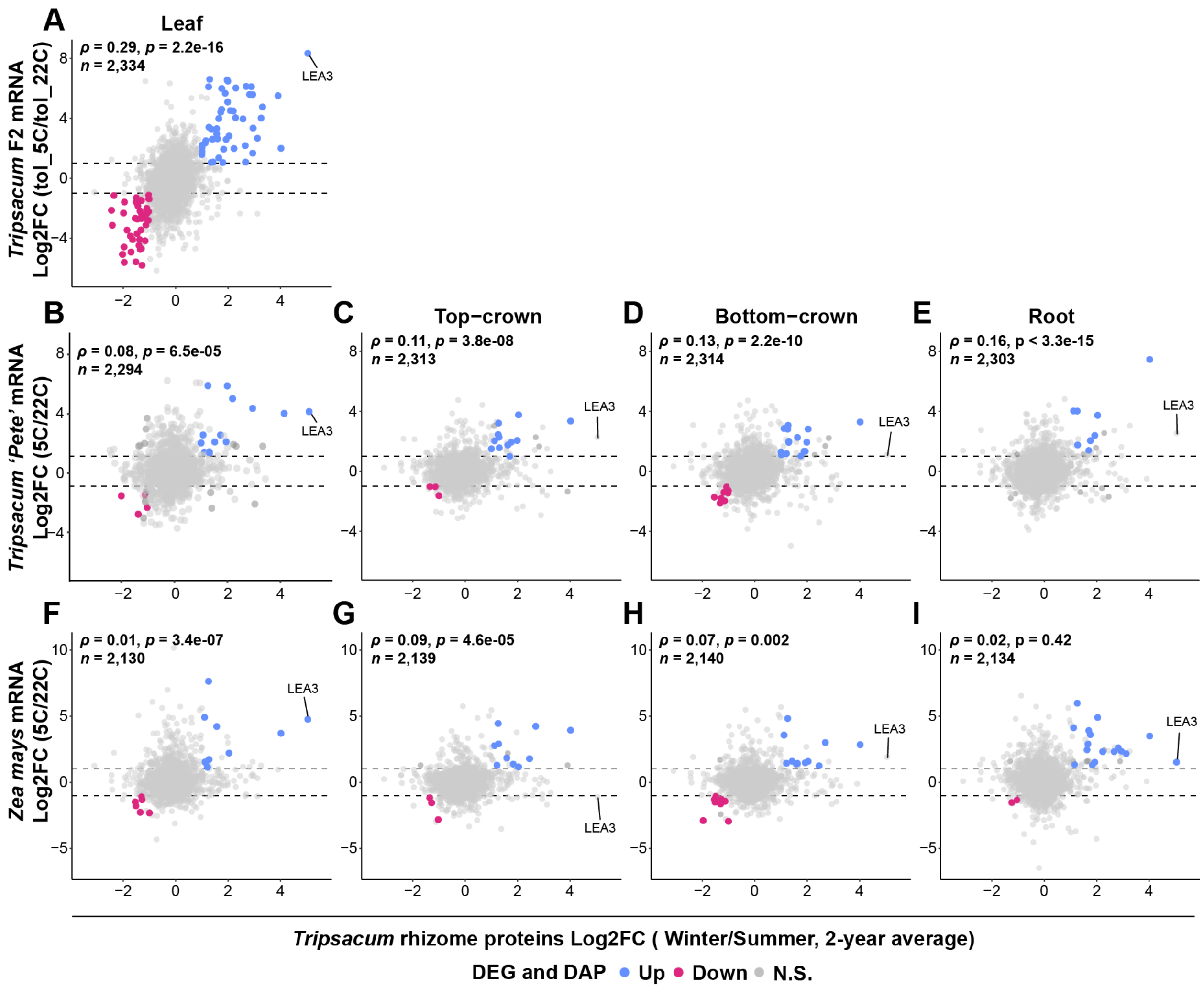

Supplemental Figure S3. **Scatterplots showing the relationship between *Tripsacum* rhizome proteins** (x-axis, log2FC Winter/Summer, 2-year average) and cold-responsive RNA expression in different seedling tissues (y-axis). **(A)** *Tripsacum* leaf mRNA from a freezing tolerant F_2 bulk before and after 7 days cold acclimation (tol_5°C/tol_22°C) descended from the same *Tripsacum* hybrids used for rhizome proteomic analysis. **(B-E)** *Tripsacum* 'Pete' tissues (5°C/22°C): leaf, top-crown, bottom-crown, and root. **(F-I)** *Zea mays* tissues (5°C/22°C): leaf, top-crown, bottom-crown, and root. Points are colored by shared differential expression status: blue for upregulated, pink for downregulated, and gray for non-significant changes. Dashed horizontal lines indicate the ±1 log2FC thresholds. Spearman's correlation coefficient (ρ) and p-value are shown in the upper left of each panel, along with the number of genes analyzed (n). The LEA3 gene is labeled in each panel.

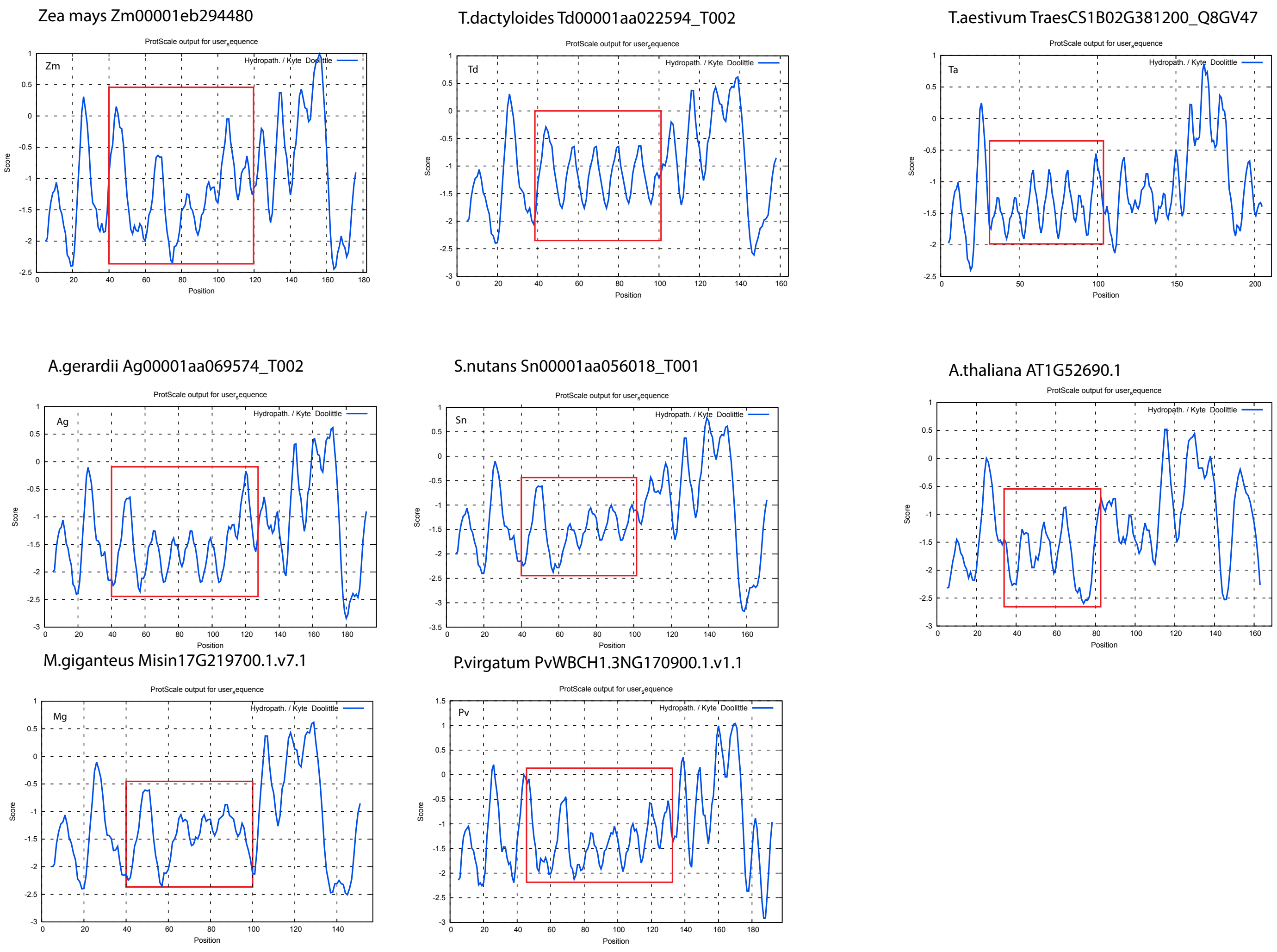

Supplemental Figure S4. Hydropathy profiles of LEA3 orthologs from eight species. Kyte-Doolittle plots were generated using ProtScale (ExPASy) with a window size of 11 amino acids. The x-axis indicates amino acid position, and the y-axis represents hydropathy score. Red boxes highlight regions corresponding to conserved 11-mer LEA motifs.

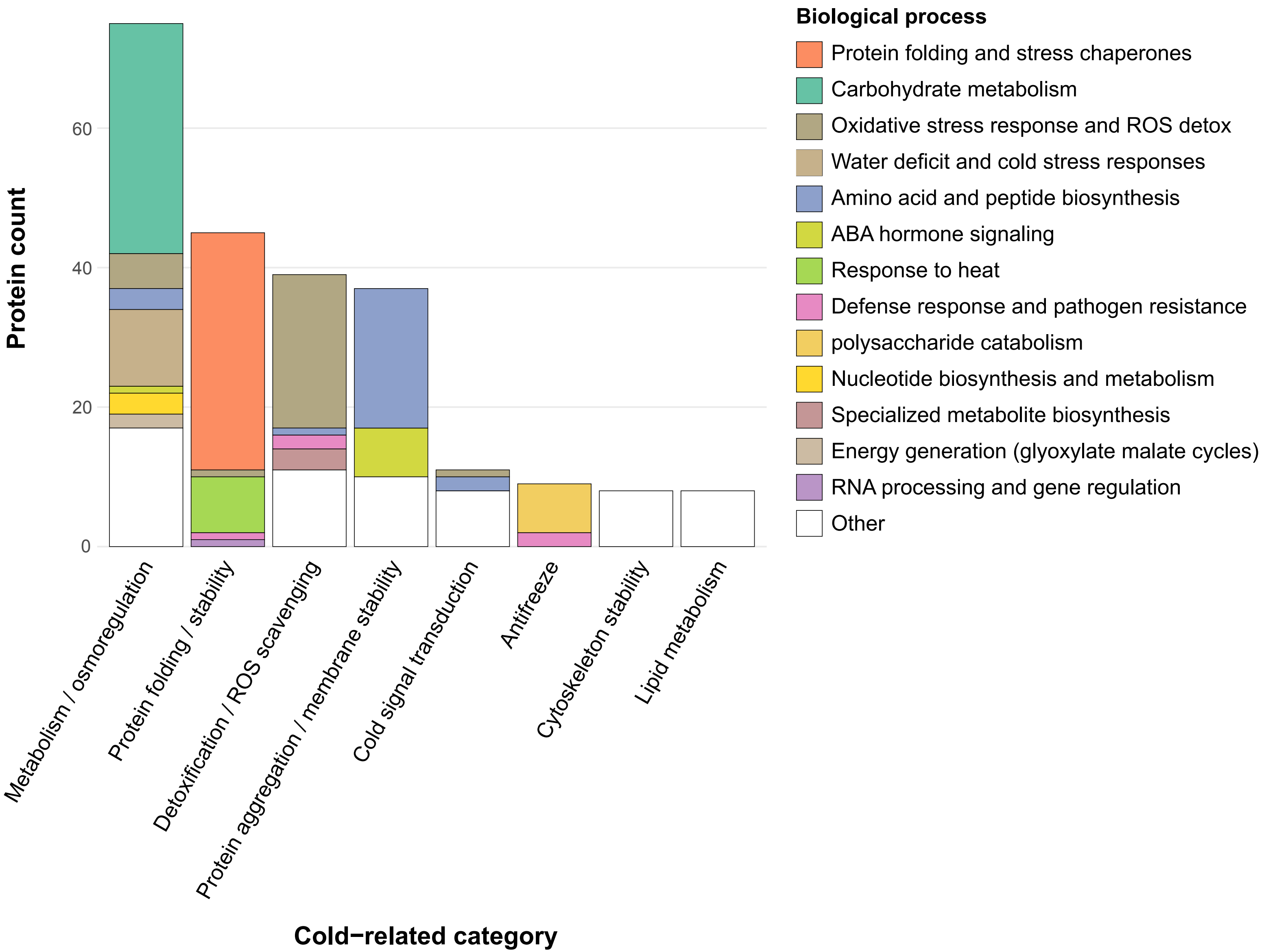

Supplemental Figure S5. Stacked bar chart illustrating the composition of BPs within curated cold-related functional categories. The plot includes 230 proteins from the top 50 DAPs upregulated in winter across species, after excluding those assigned to the cold response category "Other" (i.e., proteins with annotations deemed less relevant to cold tolerance interpretation). Cold-related categories are shown on the x-axis and sorted by total protein count. Each bar is color-coded by BP, highlighting the functional composition of each category. Less informative or poorly annotated BPs were grouped under "Other" in the legend. This visualization supports the manual consolidation of specific biological processes into broader cold-response categories used throughout the study.
